# The level of H_2_O_2_ type oxidative stress regulates virulence of Theileria-transformed leukocytes

**DOI:** 10.1111/cmi.12218

**Published:** 2013-10-21

**Authors:** Mehdi Metheni, Nadia Echebli, Marie Chaussepied, Céline Ransy, Christiane Chéreau, Kirsty Jensen, Elizabeth Glass, Frédéric Batteux, Frédéric Bouillaud, Gordon Langsley

**Affiliations:** 1 Laboratoire de Biologie Cellulaire Comparative des Apicomplexes, Faculté de Médicine, Université Paris Descartes – Sorbonne Paris CitéParis, France; 2 Inserm U1016, Cnrs UMR8104, Cochin InstituteParis, 75014, France; 3 Laboratoire d’Infections Enzootiques des Herbivores en Tunisie, Ecole Nationale de Médecine Vétérinaire, Université de la Manouba2020, Sidi Thabet, Tunisie; 4 EA 1833, Faculté de Médecine, Université Paris Descartes, Sorbonne Paris-Cité, Service d’Immunologie Biologique, Hôpital CochinParis, AP-HP, France; 5 Division of Infection and Immunity, The Roslin Institute & Royal (Dick) School of Veterinary Studies, University of Edinburgh, Easter Bush CampusMidlothian, EH25 9RG, UK; 6 Laboratoire de Mitochondries, Bioénergétique, Métabolisme et Signalisation, Faculté de Médicine, Université Paris Descartes – Sorbonne Paris CitéParis, France

## Abstract

*Theileria annulata* infects predominantly macrophages, and to a lesser extent B cells, and causes a widespread disease of cattle called tropical theileriosis. Disease-causing infected macrophages are aggressively invasive, but this virulence trait can be attenuated by long-term culture. Attenuated macrophages are used as live vaccines against tropical theileriosis and via their characterization one gains insights into what host cell trait is altered concomitant with loss of virulence. We established that sporozoite infection of monocytes rapidly induces *hif1-α* transcription and that constitutive induction of HIF-1α in transformed leukocytes is parasite-dependent. In both infectedmacrophages and B cells induction of HIF-1α activates transcription of its target genes that drive host cells to perform Warburg-like glycolysis. We propose that *Theileria*-infected leukocytes maintain a HIF-1α-driven transcriptional programme typical of Warburg glycolysis in order to reduce as much as possible host cell H_2_O_2_ type oxidative stress. However, in attenuated macrophages H_2_O_2_ production increases and HIF-1α levels consequently remained high, even though adhesion and aggressive invasiveness diminished. This indicates that *Theileria* infection generates a host leukocytes hypoxic response that if not properly controlled leads to loss of virulence.

## Introduction

The apicomplexan parasite *Theileria annulata* infects bovine macrophages and transforms them into aggressively invasive tumours that contribute to tropical theileriosis, a widespread disease endemic in North Africa, the Middle East, India and China (Dobbelaere and Heussler, 1996). In contrast to East Coast fever, caused by *T. parva*, live vaccines exist to tropical theileriosis (Darghouth, 2008) that are based on multiple-passages of infected macrophages, which become attenuated for virulence *i.e.* the infected cells have lost the heightened invasiveness virulence trait (Baylis *et al*., 1995; Hall *et al*., 1999). As the transformed state of *Theileria-*infected leukocytes can be reversed by drug-induced parasite death they represent a powerful model to study molecular events related to infection and host cell transformation in an isogenic background, e.g. the same leukocytes can be examined in the transformed and non-transformed states (Chaussepied and Langsley, 1996; Dobbelaere and Heussler, 1996). As attenuated macrophage cell-lines are directly derived from virulent ones they are also isogenic and the same infected macrophage can be examined in virulent and attenuated states. In such a way parasite-dependent TGF-β2 induction was identified as a host cell virulence trait that is lost upon attenuation (Chaussepied *et al*., 2010).

Otto Warburg observed in 1926 that cancer cells performed glycolysis even in the presence of high oxygen and that pyruvate was converted to lactate, which was secreted from the tumour, an observation that later became known as the Warburg effect (Warburg, 1956). Rapidly dividing tumour cells produce reactive oxygen species (ROS) and poor vasculature of the growing tumour results in low oxygen and hypoxia, where a major mediator of the anti-oxidant response is the hypoxia-inducible factor (HIF) family of transcription factors (Koh and Powis, 2012). HIFs are made up of three major oxygen labile subunits, HIF-1α, HIF-2α and HIF-3α and a constitutive aryl hydrocarbon receptor translocator, or ARNT/HIF-1β (Wang *et al*., 1995). Under aerobic conditions HIF-1/2α are hydroxylated by specific prolyl hydroxylases (PHDs) and hydroxylation promotes binding of an E3 ubiquitin ligase called von Hippel-Lindau (VHL) that mediates HIF degradation (Koh and Powis, 2012). During hypoxia PHD activity is diminished, VHL binding is consequently ablated and HIF is no longer degraded, leading to the accumulation of the transcription factor.

Infection by microorganisms is known to generate a host cell stress response and in *vitro* infection of human foreskin fibroblast (HFF) by *Toxoplasma gondii* provoked elevated levels of HIF-1α (Spear *et al*., 2006). As stated above, virulent *Theileria*-infected macrophages also produce TGF-β, however, they differ from *T. gondii*-infected HFFs, where intracellular growth of the parasite is arrested upon TGF-R blockade by SB505124 (Wiley *et al*., 2010). Given that *Theileria* infection of leukocytes transforms them into tumour-like cells we decided to ask whether *T. annulata* infection also generates a host cell oxidative stress response that activates HIF-1α and what are the consequences of HIF-1α induction on host cell glycolysis. We found that *hif-1α* transcription is induced within two hours of sporozoite invasion and its constitutive induction confers on infected host cells Warburg-like glycolysis, even though the cells are growing under normoxic conditions. We suggest that host leukocytes switch to Warburg glycolysis in an attempt to control toxic levels of oxidative stress stemming from *Theileria* infection and the uncontrolled host cell proliferation that ensues. We found that attenuated infected macrophages produce more H_2_O_2_ and consequently display greater signs of Warburg-like glycolysis. This observation uncouples HIF-1α induction from aggressive tumour invasiveness and implies that excessive host cell oxidative stress diminishes *Theileria*-infected macrophage virulence.

## Results

### In virulent Holstein-Friesian macrophages hif-1α induction is parasite-dependent and leads to upregulated expression of HIF-1α and its target genes

**Figure 1:**
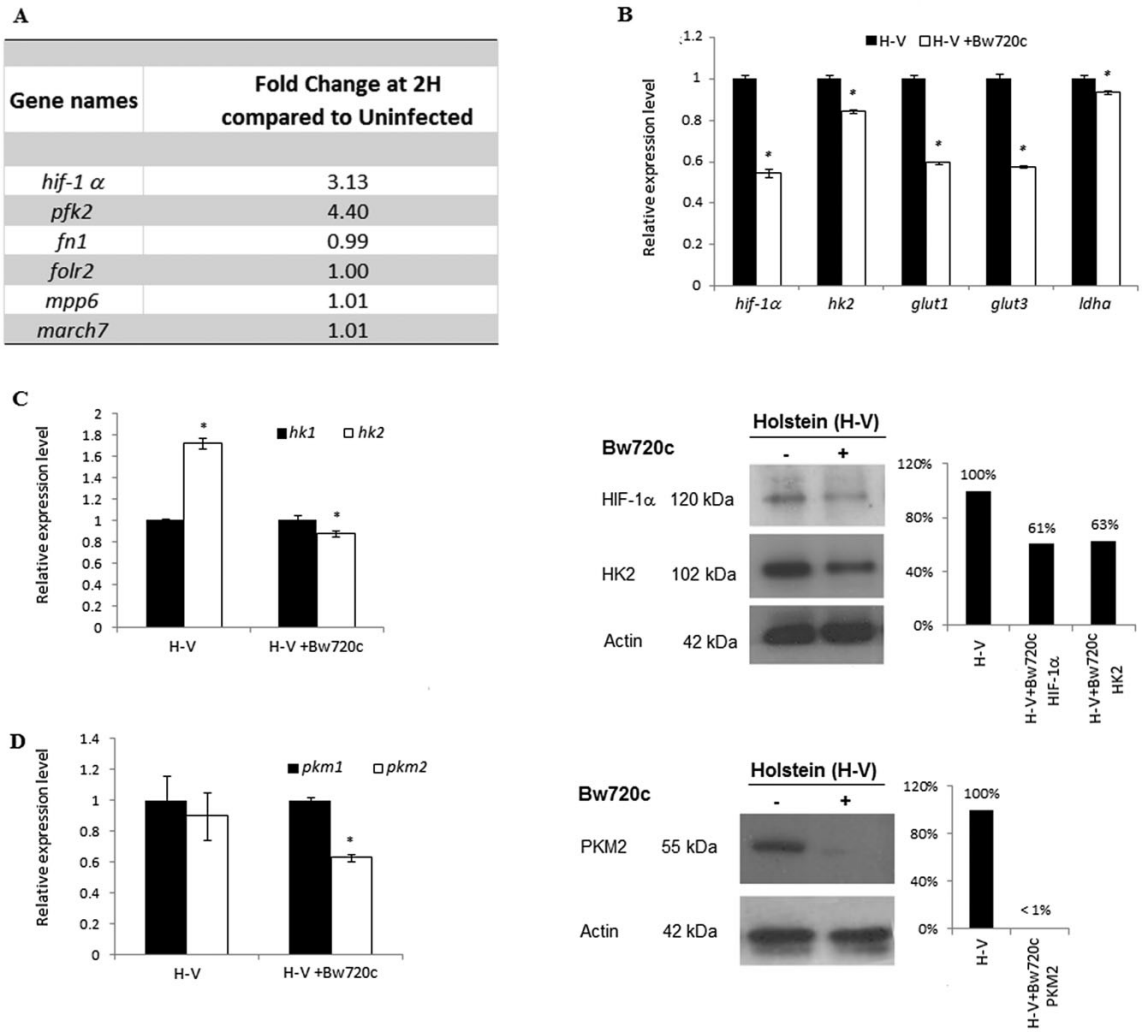
In Holstein-Friesian infected leukocytes HIF-1α activation is parasite-dependent and leads to upregulated expression of HIF-1α target genes.A. Holstein-Friesian (H)-derived resting peripheral monocytes were infected *in vitro* with *T. annulata* sporozoites and RNA was isolated and used to probe a bovine macrophage microarray. At 2 h post infection *hif-1α* transcript levels are threefold higher in infected cells compared with non-infected monocytes. The HIF-1α-target gene *pfk2* is upregulated fourfold, whereas 80% of genes show no change, including *fn1*, *folr2*, *mpp6* and *march7*.B. In virulent (H-V) macrophages the transcription levels of *hif-1α* and a selection of its target genes (*glut1*, *glut3*, *pdk1*, *ldha* and *hk2*) diminishes upon Bw720c-induced parasite death. *Student’s *t*-test, highest *P*-value = 0.0482 < 0.05.C. Bw720c-induced parasite death in H-V macrophages leads to a drop in *hk2* transcripts and a reduction in both HIF-1α and HK2 levels. *Student’s *t*-test, highest *P*-value = 0.0358 < 0.05.D. PKM2 expression also drops upon Bw720c-induced parasite death. The amount of actin expressed was used as a loading control. *Student’s *t*-test, *P*-value = 0.0376 < 0.05.

Monocytes of Holstein-Friesian (H) origin were infected *in vitro* with *T. annulata* sporozoites and RNA was isolated and used to probe a bovine macrophage microarray (Jensen *et al*., 2006; Jensen *et al*., 2008). As little as 2 h post invasion *hif-1α* transcript levels and those of a HIF-1α-target gene *pfk2* were three- to fourfold higher in H-infected monocytes compared with non-infected monocytes indicating that infection had induced *hif-1α* transcription (Fig. 1A). The transcription factor HIF-1α plays a key role in regulating the hypoxic response via transcription of a large number of target genes including those involved in glycolysis, particularly those mediating Warburg glycolysis typical of cancer cells (Warburg, 1956; Manalo *et al*., 2005; Fang *et al*., 2009). Therefore, in addition to *hif-1α* we analysed the transcription levels of four known HIF-1α-target genes: *glut1*, *glut3*, *hk2* and *ldha*, and observed that their mRNA levels decreased upon drug-induced parasite death (Fig. 1B). As Warburg glycolysis is known to promoter preferential expression of the hexokinase 2 (HK2) and pyruvate kinase 2 (PKM2) isoforms, we verified the parasite dependence of their expression compared with HK1 and PKM1 in virulent (H-V) macrophages. *T. annulata* infection of H-V induces pronounced expression of *hk2* over *hk1* and drug-induced parasite death leads to a significant reduction of both HIF-1α and HK2 proteins (Fig. 1C). Similarly, there is pronounced parasite dependence of PKM2 expression (Fig. 1D). Thus, the HIF-1α-driven programme normally associated with hypoxia-induced Warburg glycolysis depends on *Theileria*-transformed H-V macrophages harbouring live parasites.

### HIF-1α levels remain high in attenuated macrophages even as their invasiveness decreases

**Figure 2:**
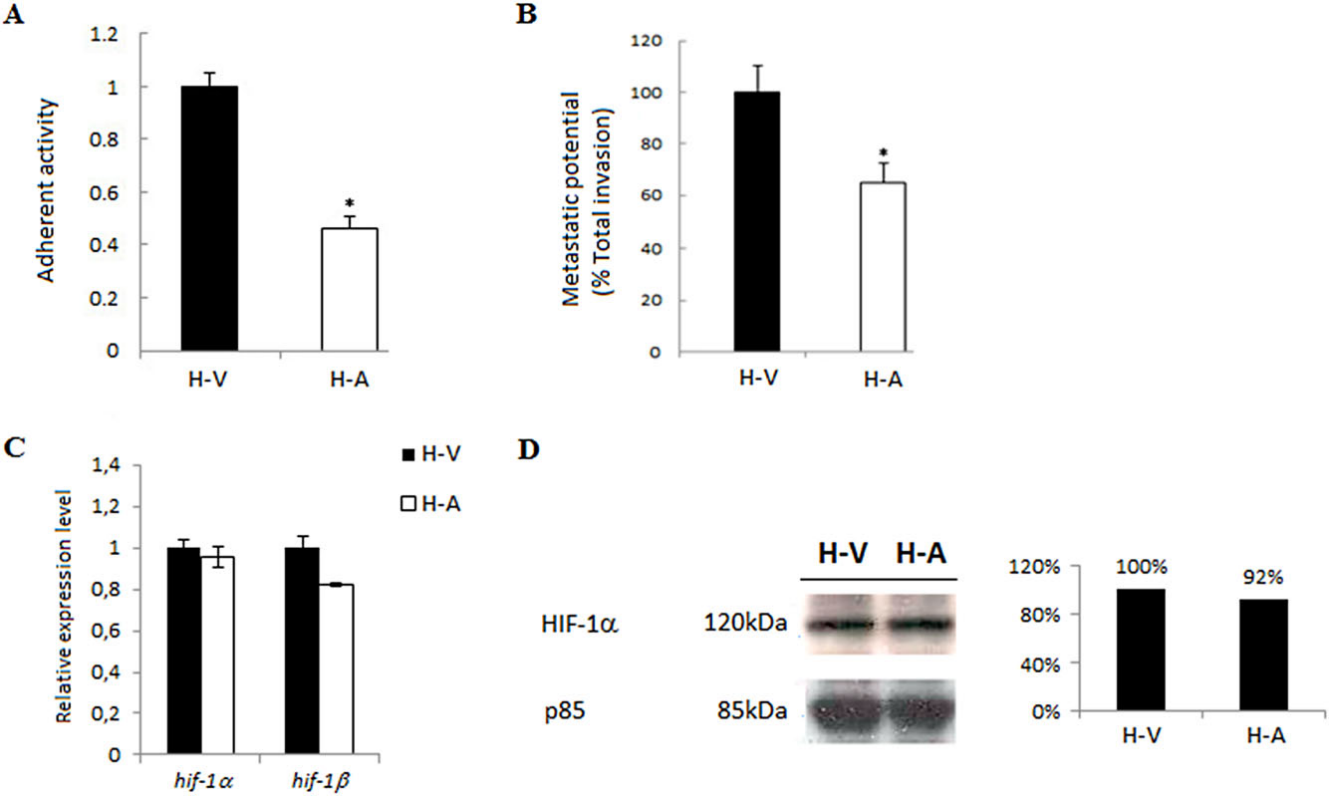
HIF-1α levels remain high in attenuated macrophages even as adhesion and invasiveness decreases.A. Virulent Holstein-Friesian macrophages (H-V) adhere to fibronectin and binding is significantly reduced in attenuated (H-A) macrophages even though attenuated cell spread more than virulent ones.B. H-V macrophages degrade Matrigel better than H-A macrophages.C. Transcription of *hif-1α* and *hif-1β* is unchanged in H-V macrophages compared H-A macrophages. Transcript levels were normalized to *hprt1* expression. *Student’s *t*-test, NS *P*-value > 0.05.D. HIF-1α protein levels also remain largely unchanged in H-V and H-A macrophages. The level of p85 expression was used as a loading control.

Virulent Holstein-Friesian (H-V) *T. annulata*-infected macrophages can be attenuated (H-A) by long-term culture and are used to vaccinate against tropical theileriosis. As well as promoting Warburg glycolysis, HIF induction also contributes to tumour aggressiveness due to its capacity to promote transcription of genes involved in angiogenesis, for recent reviews see (Levine and Puzio-Kuter, 2010; Koh and Powis, 2012). As H-A macrophages lose both their adhesive (Fig. 2A) and invasive capacities (Fig. 2B), we expected that these cells would exhibit decreased expression of *hif-1α* and *hif-1β*. Surprisingly, *hif-1α* and *hif-1β* mRNA levels were largely unchanged (Fig. 2C). Attenuation of *Theileria*-transformed macrophages and their corresponding loss of tumorigenicity are therefore independent of HIF levels that remain high (Fig. 2D).

### *Theileria*-transformed macrophages display signs of aerobic glycolysis (Warburg effect)

**Figure 3:**
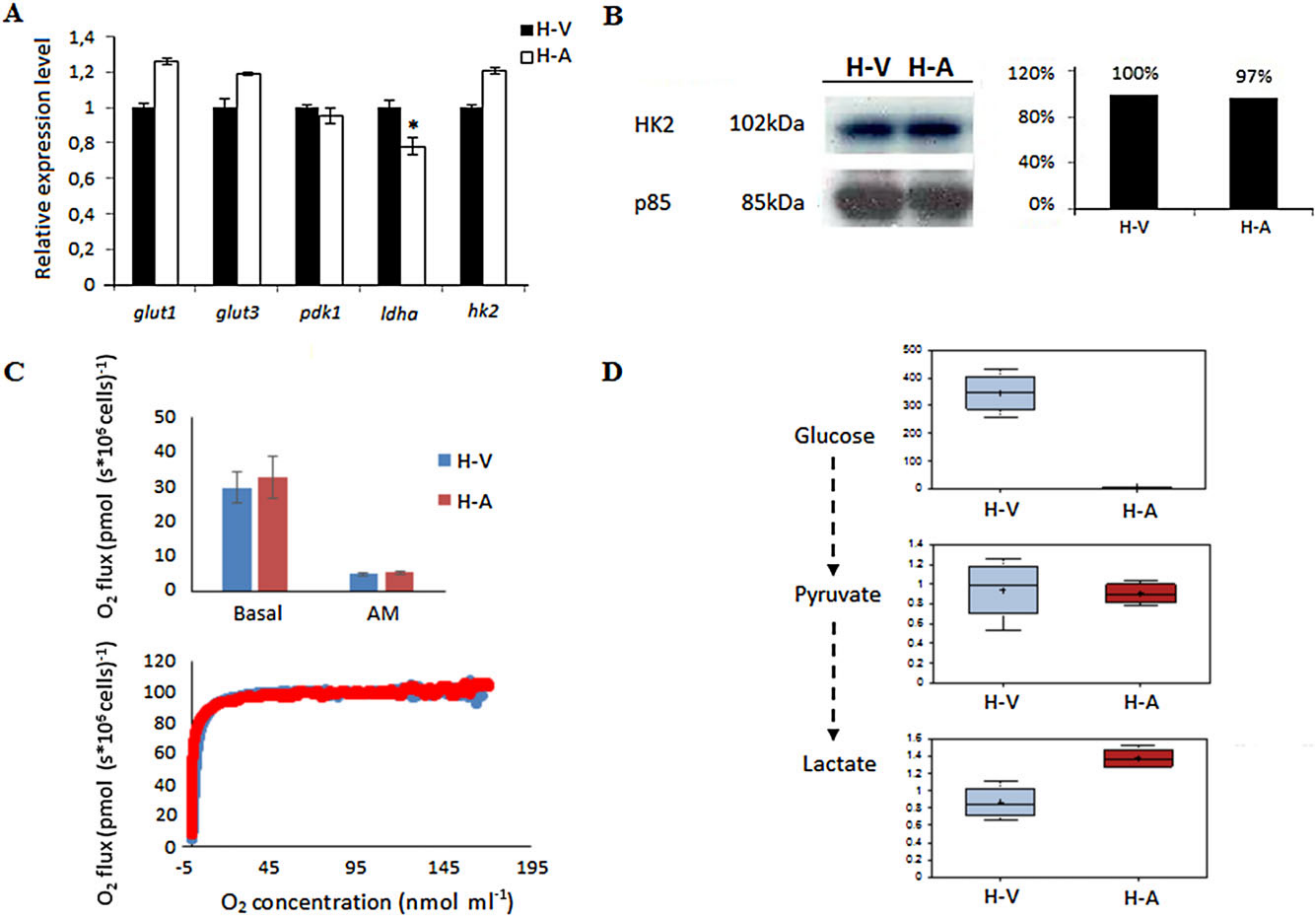
HIF-1α-target gene expression in the presence of high O_2_ consumption and lactate output.A. Transcription levels of five *hif-1α* target genes; *glut1*, *glut3*, *pdk1*, *ldha* and *hk2*, were investigated in virulent (H-V) and attenuated *T. annulata*-infected (H-A) macrophages. Only *ldha* levels are lower in H-A macrophages. *Student’s *t*-test, highest *P*-value = 0.0074 < 0.05.B. HK2 protein levels remained equivalent in H-V and H-A macrophages. The amounts of p85 were used as a loading control.C. Upper panel, three independent Oroboros measurements show O_2_ fluxes are equivalent in both H-A and H-V macrophages and respiration is largely mitochondrial, since upon antimycin (AM) inhibition of complex III O_2_ flux is markedly decreased. Lower panel, O_2_ consumption over time is the same in H-V and H-A macrophages in a closed Oroboros chamber, which eventually becomes hypoxic. Between each experiment Oroboros chambers were washed 3 times and incubated for at least 5 min with ethanol then rinsed with water.D. Metabolomic analyses highlighting the intracellular metabolite levels stemming from glycolytic activity of H-V and H-A macrophages. H-A macrophages consume more glucose and produce more lactate than H-V macrophages, whereas pyruvate levels are equivalent.

As neither *hif-1α* nor *hif-1β* levels changed significantly the expression of a selection of HIF-target genes also remained upregulated in attenuated *T. annulata*-infected macrophages, with only *ldha* transcription being somewhat dampened (Fig. 3A). Attenuated H-A infected macrophages maintain HK2 protein levels (Fig. 3B), consume oxygen (Fig. 3C), consume glucose and produce lactate (Fig. 3D), all traits typically associated with Warburg type glycolysis. Greater glucose consumption with higher lactate output is also true for a B-cell line (BL3) infected with *T. annulata* (TBL3, Fig. S1C). Taken together, all data concur and suggest that following invasion *T. annulata*-transformed leukocytes perform Warburg-like glycolysis.

### Infection induces H_2_O_2_ type oxidative stress, and attenuation of virulence increases H_2_O_2_ output

**Figure 4:**
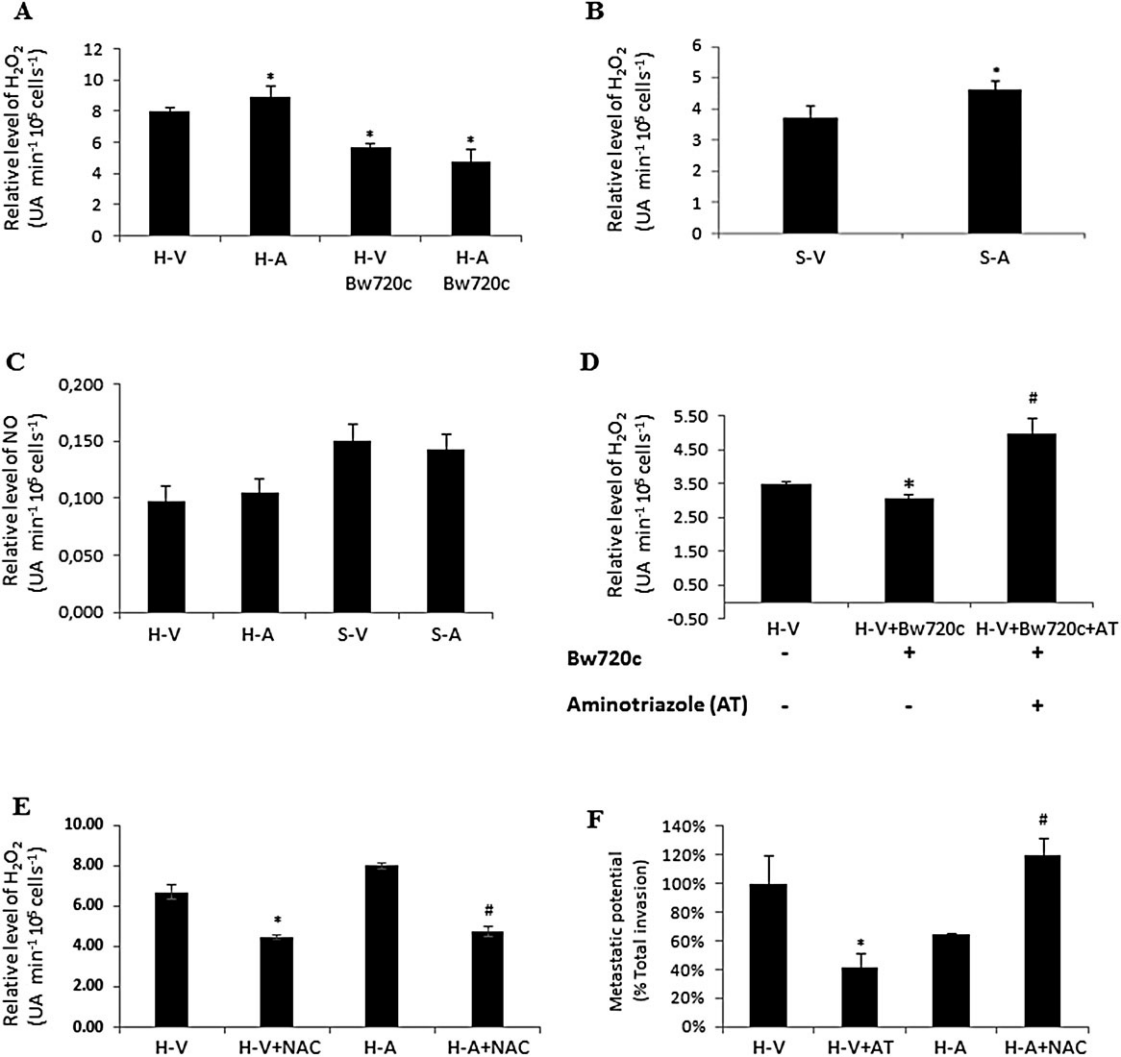
Infection induces H_2_O_2_ type oxidative stress and attenuation increases H_2_O_2_ output.A. Virulent (H-V) *T. annulata*-infected macrophages produce less H_2_O_2_ than attenuated (H-A) *T. annulata*-infected macrophages and the amount of H_2_O_2_ is reduced upon Bw720c-induced parasite death.B. Virulent Sahiwal/Holstein-Friesian (S-V) *T. annulata*-infected macrophages produce less H_2_O_2_ than attenuated (S-A) macrophages.C. The levels of NO are unchanged between virulent and attenuated *T. annulata*-infected macrophages of H and S origin, even though Sahiwal/Holstein-Friesian infected macrophages produce more NO.D. Bw720c does not incapacitate macrophage production of H_2_O_2_ when host cells are stressed by AT treatment.E. The anti-oxidant NAC reduces H_2_O_2_ output by H-V and H-A macrophages.F. Increasing H_2_O_2_ output by AT blockade of catalase reduces transwell migration by H-V and conversely, reducing H_2_O_2_ levels with NAC increases transwell migration of H-A macrophages. #, *Student’s *t*-test, highest *P*-value = 0.04 < 0.05.

Figures 3 show that HIF-1α, HK2 and other HIF-1α-target genes involved in aerobic glycolysis are expressed even though *Theileria*-infected H-V and H-A macrophages are consuming O_2_ and growing under normoxic conditions. As oxidative stress has been linked to HIF-1α activation (Bonello *et al*., 2007; Pialoux *et al*., 2009), we measured the H_2_O_2_ output of *T. annulata*-infected macrophages of different origins (Fig. 4). Attenuated macrophages of pure breed (H-A), or cross-breed (S-A) origin produced more H_2_O_2_ compared with NO, than their virulent counterparts (Fig. 4A–C). H_2_O_2_ output from macrophages is parasite-dependant, as it diminishes on Bw720c treatment (Fig. 4A). Similarly, H_2_O_2_ levels are also significantly higher in infected TBL3 compared with non-infected BL3 lymphocytes (Fig. S1C). Bw720c-induced parasite death does not diminish the capacity of the host macrophage to produce H_2_O_2_, as output is sustained by aminotriazole (AT) inhibition of macrophage catalase that converts H_2_O_2_ to 2 H_2_O (Fig. 4D). Consistently, the antioxidant NAC reduces H_2_O_2_ output even in the presence of live parasites (Fig. 4E). Reducing high H_2_O_2_ output by attenuated H-A macrophages increases their transwell migration and conversely, increasing H_2_O_2_ output by H-V macrophages dampens their transwell migration (Fig. 4F). *Theileria* infection of macrophages therefore induces H_2_O_2_ type oxidative stress that underpins their invasive capacity.

### H_2_O_2_ output by infected macrophages regulates HIF-1α levels

**Figure 5:**
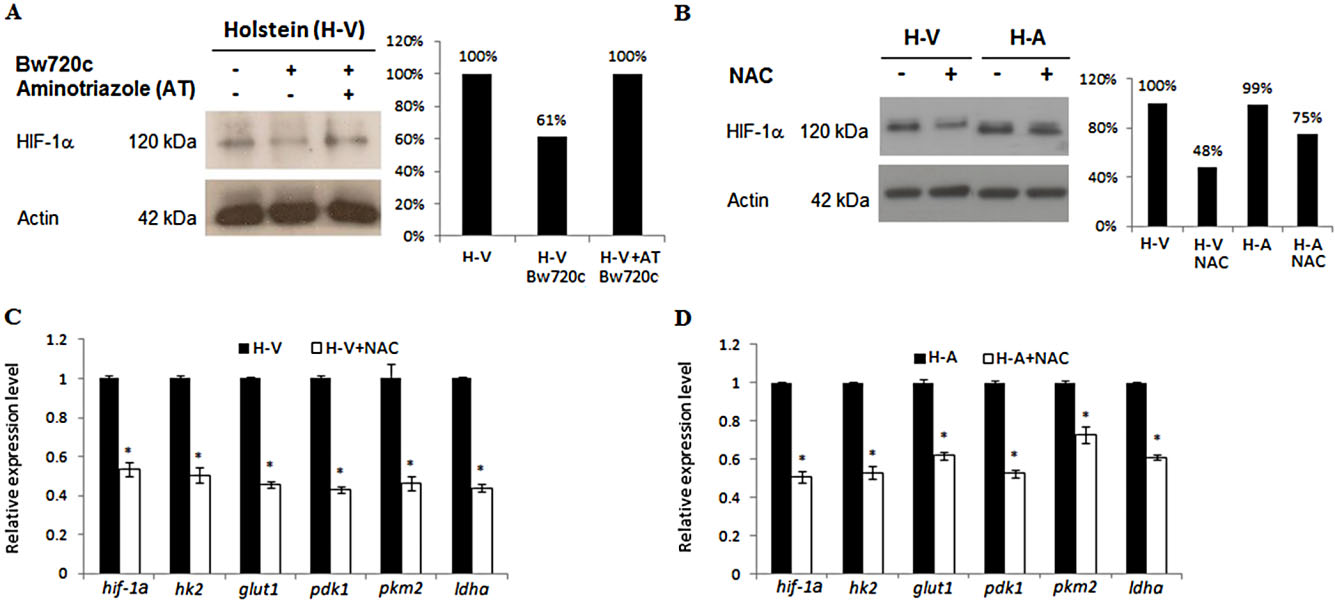
H_2_O_2_ output by infected macrophages regulates HIF-1α levels.A. Bw720c treatment reduces HIF-1α levels, but does not inhibit the host cell HIF-1α response when H-V macrophages are stressed by aminotriazole (AT) treatment.B. Lowering H_2_O_2_ levels by NAC treatment also reduces HIF-1a expression in both H-V and H-A macrophages.C. NAC treatment of H-V reduces the expression of *hif-1α* and 4 different *hif-1α*-target genes.D. Similarly, NAC treatment of H-A reduces the expression of *hif-1α* and 4 different *hif-1α*-target genes. *Student’s *t*-test, highest *P*-value = 0.013 < 0.05.

In Figs 1 and 4 we show that HIF-1α levels and H_2_O_2_ output depend on live parasites raising the question as to whether it is the parasite, or H_2_O_2_ that is responsible for HIF-1α expression. The parasite was killed by Bw720c treatment and H_2_O_2_ output sustained by AT inhibition of catalase and under these conditions the level of HIF-1α expression is maintained (Fig. 5A). It follows that reducing H_2_O_2_ by NAC treatment also diminishes HIF-1α expression (Fig. 5B) and consequently HK2 levels (data not shown). Consistently, NAC treatment also reduced transcription of HIF-target genes in both H-V and H-A macrophages (Fig. 5C and D). Clearly, it is H_2_O_2_ that induces the HIF-1α-driven Warburg-like programme of parasite-transformed macrophages.

### Loss of HF-1α provokes a shift to more oxidative phosphorylation and reduced macrophage proliferation

**Figure 6:**
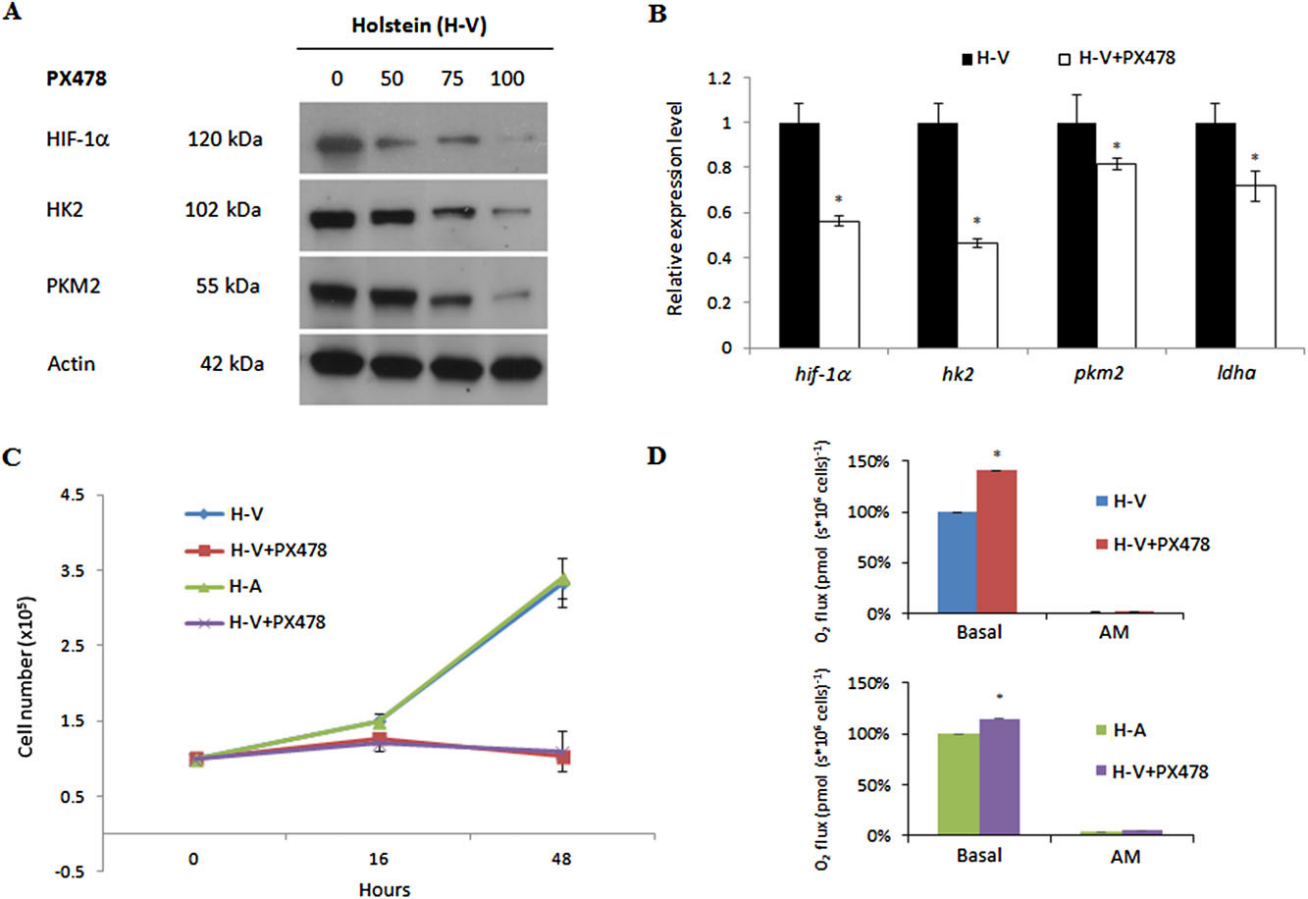
Loss of HIF-1α provokes a shift to more oxidative phosphorylation and reduced macrophage proliferation.A. 16 h treatment of H-V macrophages with increasing concentrations of PX478 results in reduced levels of HIF-1α, HK2 and PKM2 compared with actin.B. Treatment with PX748 at 100 μM for 16 h reduces expression of *hif-1a*, *hk2*, *pkm2* and *ldha*.C. 16 h of PX748 at 100 μM initiates a proliferation arrest that becomes pronounced at 48 h.D. Three independent Oroboros measurements of both H-A and H-V macrophages treated for 16 h with PX748 show O_2_ fluxes increase and respiration is more mitochondrial, as upon antimycin (AM) inhibition of complex III O_2_ flux is strongly diminished. *Student’s *t*-test, highest *P*-value = 0.03395 < 0.05.

To directly link HIF-1α to HK2, PKM2 and LDHA expression, infected Holstein-Friesian macrophages were treated with increasing doses of the specific HIF-1α inhibitor PX478 (Fig. 6). HIF-1α levels were progressively lost with increasing concentrations of PX478 and consequently HK2 and PKM2 diminished according (Fig. 6A). Complete loss of HIF-1α occurred after treatment with 100 μM PX478 for 16 h and this reduced transcription of *hif-1α*, *hk2*, *pkm2* and *ldha* (Fig. 6B) and initiated an arrest of infected macrophage proliferation and prolonged inhibition leads to a complete block in growth (Fig. 6C). Upon PX478-induced loss of HIF-1α both H-V and H-A macrophages consume more oxygen indicating that they are more OXPHOS-like (Fig. 6C).

## Discussion

We have shown that monocyte invasion by *T. annulata* sporozoites activates *hif-1α* transcription and that HIF-1A remains induced even as transformed macrophage virulence is lost. However, it is possible that the pathways leading to HIF-1α activation immediately following invasion differ slightly to those that maintain HIF-1α induction in established *Theileria*-transformed cell lines. The *hif-1a* promoter is known to have binding sites for HIF-1α, NF-κB and AP-1 (Frede *et al*., 2007). As both NF-κB and AP-1 are induced by *Theileria* infection (Palmer *et al*., 1997; Botteron and Dobbeldeare, 1998; Chaussepied *et al*., 1998), it is likely that these two transcription factors participate in the rapid induction of *hif-1α* transcription post sporozoite invasion. Once induced HIF-1α can contribute to maintaining its own transcription and the loss of *hif-1α* message upon PX478 inhibition of HIF-1α (Fig. 6) is entirely consistent with this notion. Indeed, this may compensate for the loss of AP-1-driven transcription that characterizes attenuated vaccine lines (Baylis *et al*., 1995; Adamson *et al*., 2000).

We examined also the expression profiles of 6 genes (*glut1*, *glut3*, *hk2*, *pdk1*, *ldha* and *pkm2*) known to be under the control of HIF-1α (Keith *et al*., 2012). This showed that *Theileria* infection has activated a leukocyte transcriptional programme typical of Warburg glycolysis (e.g. higher expression of *hk2* and *pkm2*), which is normally associated with cells experiencing hypoxia (Levine and Puzio-Kuter, 2010). This was unexpected for *Theileria*-infected macrophages that are consuming O_2_ (Fig. 3C). We therefore performed global metabolomic analyses on virulent (H-V) compared with attenuated (H-A) *T. annulata*-infected macrophages cells and compared the levels of intracellular metabolites associated with HK2, PKM2 and LDHA (Fig. 3D). This confirmed that *Theileria* infection confers on its host leukocyte (macrophage and B cell) high glucose consumption and lactate output (only intracellular lactate was measured so the levels in Fig. 3D and Fig. S1D do not account for secreted lactate), characteristics typical of Warburg-like glycolysis. We considered that the metabolome of infected TBL3 cells is composed of metabolites coming not only from leukocyte glycolysis, but also from the parasite glycolytic pathway, as apicomplexan parasites are known to produce abundant lactate (Ginsburg, 2006). However, HK2 expression is typical of mammalian cells performing Warburg glycolysis (Wolf *et al*., 2011) and only infected leukocytes express HK2 (see Fig. S1B).

Attenuated *T. annulata*-infected macrophages are used as live vaccines against tropical theileriosis, because the cells have lost their virulent hyper-invasiveness (Hall *et al*., 1999). When we examined virulent compared with attenuated *T. annulata*-infected macrophages we expected to find reduced HIF-1α levels, since HIF-1α activation is frequently associated with aggressive tumours and poor prognosis (Jubb *et al*., 2010). Instead, HIF-1α levels were unchanged in attenuated infected macrophages and consequently HK2 levels also remained constant (Fig. 2D). Since, expression of HIF-1α and its target-genes remained high, even as infected macrophages lose virulence, we compared production and consumption of glycolysis metabolites between virulent and attenuated infected macrophages (Fig. 3D). Attenuated macrophages consume more glucose and produce more lactate than virulent infected macrophages indicating that upon attenuation glycolytic activity increased *i.e.* in spite of being attenuated the cells have become more Warburg-like. Since the virulent versus attenuated metabolomes were derived on the same background (the same Holstein-Friesian macrophages infected with *Theileria annulata*), the differences observed are due to host cell metabolites, as live intracellular parasites are always present.

Combined, the data indicate that *T. annulata* infection drives its host cell to perform Warburg-like glycolysis even though the 2 different infected (H and S) macrophage lines examined here are proliferating under normoxic conditions. Why *Theileria*-infected macrophages should maintain a HIF-1α-driven transcriptional programme typical of Warburg glycolysis is curious, but as infection induces *hif-1α* transcription within two hours of sporozoite invasion it is conceivable that the infected host cell switches to Warburg-like glycolysis in order to keep the level infection-induced ROS under toxic levels. However, a few *Theileria*-infected cell lines like TBL3 grow in suspension as clusters that could result in them experiencing hypoxia that would contribute to HIF-1α activation. Indeed, a recent TBL3-based study showed that HIF-1α inhibition led to a reduced formation of colonies in soft agar (Medjkane *et al*., 2013), whereas as in attenuated macrophages HIF-1α is maintained even though virulence is lost. This implies that in attenuated *T. annulata*-infected macrophages HIF-1α is driving a transcriptional programme unrelated to transformed leucocyte invasiveness, such as Glut1-mediated glucose uptake necessary for infected leucocyte proliferation. Specific PX478 inhibition of HIF-1α confirmed its role in macrophage proliferation (Fig. 6C) and it is possible that the failure of TBL3 cells to form colonies in soft agar upon HIF-1α inhibition (Medjkane *et al*., 2013) is associated with a proliferation defect. Comparing the HIF-1α transcriptional programmes of virulent compared with attenuated *T. annulata*-infected macrophages should allow the identification of the subset of HIF-1α-target genes regulating hyper invasiveness of *Theileria*-transformed macrophages.

We have shown that two independent attenuated macrophage lines are more stressed their virulent counterparts. Attenuation is concomitant with loss of JNK activity (Chaussepied and Langsley, 1996; Galley *et al*., 1997; Chaussepied *et al*., 1998) and JNK activation can mediate an anti-oxidant response (Shen and Liu, 2006; Fang *et al*., 2009). Loss of JNK activity might render attenuated macrophages less capable of controlling oxidative stress and hence, they produce more H_2_O_2_. Furthering our understanding of how varying levels of H_2_O_2_ output is linked to *Theileria*-induced virulence and eventual attenuation will stimulate future studies.

## Experimental procedures

### *Theileria*-infected cell lines

Holstein-Friesian monocytes were prepared and infected with *T. annulata* sporozoites, as described in Jensen *et al*. (2008). The characterization of the Holstein-Friesian Ode vaccine line from India has been reported previously (Singh, 1990). In this study virulent Holstein-Friesian (H-V) corresponds to Ode passage 61–70 and attenuated (H-A) to Ode passage 309–322. BL3 and TBL3 cells used in this study have been characterized (Theilen *et al*., 1968; Moreau *et al*., 1999). In this study the virulent Sahiwal/Holstein-Friesian crossbreed-derived *T. annulata*-infected cell-line (S-V) corresponds to cloned Jed4 passage 18 and the attenuated cell-line (S-A) to cloned Jed4 passage 333 (Darghouth *et al*., 1996). All cultures were maintained in RPMI-1640 medium supplemented with 10% fetal calf serum (FCS), 100 μg ml^−1^ penicillin, 100 UI ml^−1^ streptomycin, 2 mM l-glutamine, 10 mM Hepes and 5% 2-mercapthoethanol for BL3 and TBL3. Holstein-Friesian cells were treated 72 h with the drug buparvaquone (Bw720c) to eliminate the parasite as described in Lizundia *et al*. (2006); 24 h with 3-Amino-1,2,4-triazole (Sigma A8056-10G) to block catalase activity (Bayliak *et al*., 2008; Walton and Pizzitelli, 2012); 24 h with *N*-acetylcysteine (NAC, Sigma) and for up to 16 h with PX478 to inhibit HIF-1α (MedKoo Biosciences).

### Western blot analysis

After indicated treatments cells were harvested and were extracted with lysis buffer (Hepes 20 mM, pH 8; NaCl 150 mM; EDTA 2 mM; Nonidet P40 1%; SDS 0.1%; Sodium deoxycholate 0.5% containing proteases inhibitors (Complete mini EDTA free, Roche) and phosphatase (PhosSTOP, Roche). Lysates were centrifuged at 13 000 r.p.m. for 15 min at 4°C, and supernatants collected. Equal amounts of protein were separated by SDS-PAGE, transferred to nitrocellulose membrane (Protran, Whatman) at 30 V overnight at 4°C and blocked with 4% skimmed milk for 1 h. The antibodies used in immunoblotting were as follows: anti-HIF-1α (Abcam, ab2185); anti-HK2 (Cell Signaling, #2867S); Anti-PI3-K (p85) antibody (Merck Millipore, 06-195); anti-PKM2 (Cell Signaling #3198S) and anti-actin (I-19, Santa Cruz Biotechnology).

### Total RNA extraction

Total RNA was isolated from each of the *T. annulata*-infected cell lines using the RNeasy mini kit (Qiagen) according to the manufacturer’s instructions. The quality and quantity of the resulting RNA was determined using a Nanodrop spectrophotometer. mRNA was reverse transcribed to first-strand cDNA and the relative levels of each transcript were quantified by real-time PCR using SYBR Green detection. The detection of a single product was verified by dissociation curve analysis and relative quantities of mRNA calculated using the method described by (Pfaffl, 2001). *Hprt1* was used to normalize mRNA levels.

Primer sequences use for qRT-PCR:

**Table:** 

	Sense	Antisense
*Hif-1a*	GCTTGCTCATCAGTTGCCAC	AGCTGATGGTGAGCCTCATA
*Hif-1b*	GGAACCACGACCTTCACTGT	GCCCATCTCCAGGGATAAAT
*Glut1*	TCTCCGTGGGCCTTTTTGTT	AGGCCAGCAGGTTCATCATC
*Glut3*	ACTTTGGAAGAGCGGTCAGA	AAGGACCACAGGGATGTGAG
*Hk1*	GGGACGCTCTACAAGCTTCA	CAGTTCCTTCACGGTTTGGT
*Hk2*	CCGGGAAGCAACTATTTGAA	TCACCAGGATAAGCCTCACC
*Pdk1*	CCGCCTATTCAGGTCCATGT	ACCTCCTCGGTCACTCATCT
*Pkm1*	CCTGATAGCTCGTGAGGCTG	GCTCGCGCAAGTTCTTCAAA
*Pkm2*	GGAATGAATGTGGCTCGTCT	ATGGTCTCCGCATGGTACTC
*Ldha*	GTTGCTGGTGTCTCCCTGAA	TGTGAACCGCTTTCCACTGT

### Intracellular levels of hydrogen peroxide (H_2_O_2_)

Holstein-Friesian (H) *T. annulata*-infected macrophages (1 × 10^5^ cells per well) were seeded in 96-well plates and incubated 18 h in complete medium. Cells were washed in PBS and incubated with 100 μl per wells of 5 μM H2-DCFDA diluted in PBS (Molecular Probes). H_2_O_2_ levels were assayed by spectrofluorimetry on a fusion spectrofluorimeter (PackardBell). Fluorescence intensity was recorded every hour over a period of 5 h. Excitation and emission wavelengths used for H_2_O_2_ were 485 and 530 nm. The number of cells was evaluated by the crystal violet assay. Cells were stained in 0.05% crystal violet and 2% ethanol in PBS for 30 min at room temperature. After four washes in PBS, the stain was dissolved in methanol and measured at 550 nm on Fusion. The level of H_2_O_2_ was calculated in each sample as follows: reactive oxygen species rate (arbitrary units min^−1^ 10^5^ cells^−1^) = [fluorescence intensity (arbitrary units) at T300 minutes − fluorescence intensity (arbitrary units) at T0] per 60 min per number of cells as measured by the crystal violet assay.

### Adhesion assay

A 96-well plate was coated with bovine fibronectin (Sigma #F1141), 2 μg cm^−2^ diluted in double distilled water overnight at 4°C. The plate was then washed twice with 100 μl 0.1% BSA in RPMI-1640 and blocked for 1 h at 37°C by 0.5% BSA in RPMI-1640. After two washes, 1 × 10^4^ cells were added to each well and incubated at 37°C, 5% CO_2_ for 30 min. Non-adherent cells were removed by washing the wells three times before fixing with 100 μl 4% paraformaldehyde for 10 min at room temperature. Following one further wash, wells were stained with 100 μl crystal violet (1 mg ml^−1^) for 10 min at room temperature. Wells were extensively washed with distilled water and air-dried. Samples were re-suspended by 30 min incubation at room temperature in 100 μl 2% SDS, 2% ethanol before reading the optical density at 595 nm.

### Invasion assays of H-transformed cells

The invasive capacity of the Holstein-Friesian *T. annulata*-infected macrophage cell-line was assessed *in vitro* using Matrigel migration chambers: culture Coat 96 well medium BME cell invasion assay was obtained from Culturex Instructions (3482-096-K). After 24 h of incubation at 37°C, the top chamber was washed once in buffer and placed back on the receiver plate. One hundred microlitres of cell dissociation solution/Calcein AM was added to the bottom chamber of each well, incubated at 37°C for 1 h to fluorescently label cells and dissociate them from the membrane before reading at 485 nm excitation, 520 nm emission using the same parameters as the standard curve.

### Metabolome data

Virulent and attenuated *T. annulata*-infected H macrophages together with BL3 and TBL3 B cells were prepared according to the Metabolon (http://www.metabolon.com/) protocol. Briefly, leucocytes were flash frozen and shipped on dry ice to Metabolon for profiling. The analyses were performed in quadruplicate and 334 different metabolites were detected. Intracellular metabolites stemming from glycolytic activity associated with hexokinase, pyruvate kinase and lactate dehydrogenase were extracted from the global metabolome for this purpose of this study.

### Oroboros measurements of O_2_ consumption

O_2_ concentration and consumption by H-V and H-A infected macrophages was measured by a high-resolution respirometer (Oroboros Oxygraph-2k). Both electrodes were calibrated at 37°C and 100% oxygen before adding 2.5 ml of cells (2 × 10^6^ cells ml^−1^) to each chamber. Antimycin was added at the basal level to block complex III, as an estimate of mitochondrial contribution to overall cell respiration. When the chambers are left closed, cells gradually consume all the O_2_ present in the media until they become hypoxic and consequently the flux of O_2_ consumption decreases to 0 [pmole (s*ml)^−1^].

## Supporting Information

**Fig S1** Infected BL3 lymphocytes display signs of aerobic glycolysis. A. *T. annulata*-infected TBL3 cells express HIF-1α to greater levels than non-infected BL3 lymphocytes. Actin amounts were used as a loading control. B. *T. annulata* infection strongly induces HK2 expression in TBL3 lymphocytes, whereas as in non-infected BL3 cells HK2 is below the level of detection. Actin was used as a loading control. C. Non-infected BL3 lymphocytes produce less H_2_O_2_ than infected TBL3 lymphocytes. D. Metabolomic analyses highlighting intracellular metabolites stemming from glycolytic activity associated with hexokinase, pyruvate kinase and lactate dehydrogenase in BL3 compared with TBL3 lymphocytes. TBL3 consume more glucose and produce more lactate than BL3, whereas pyruvate levels remain approximately equivalent. The intracellular lactate levels do not take into account lactate secreted into the culture medium.
