# Supplementary figures and images for: The level of H2O2 type oxidative stress regulates virulence of Theileria-transformed leukocytes
Source: Cell Microbiol. 2013 Oct 21;16(2):269–79. doi: 10.1111/cmi.12218 (PMC3906831; doi:10.1111/cmi.12218)

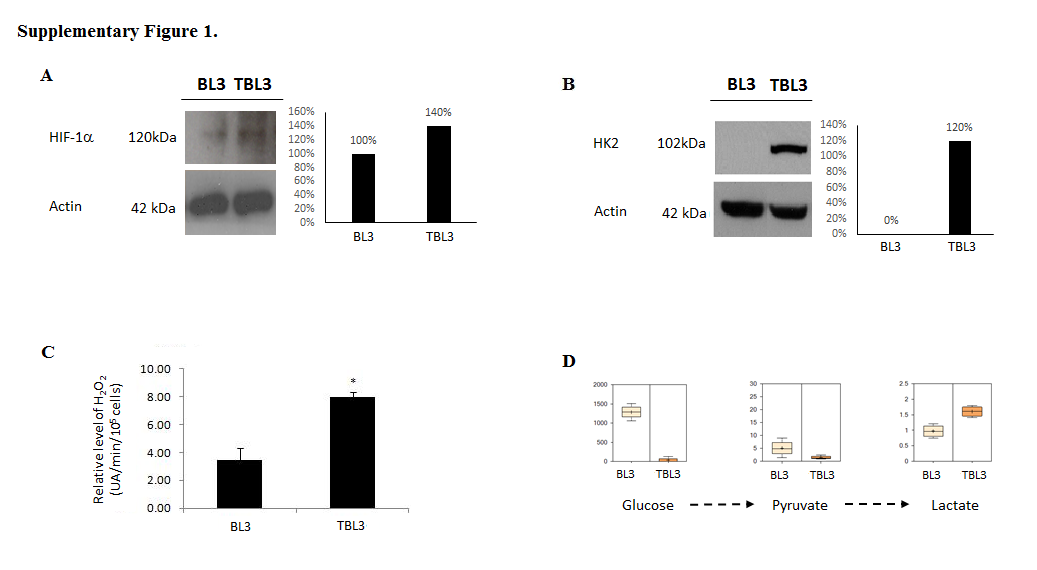

Supplement: Supplementary file 1 — Fig S1 Infected BL3 lymphocytes display signs of aerobic glycolysis. A. T. annulata-infected TBL3 cells express HIF-1α to greater levels than non-infected BL3 lymphocytes. Actin amounts were used as a loading control. B. T. annulata infection strongly induces HK2 expression in TBL3 lymphocytes, whereas as in non-infected BL3 cells HK2 is below the level of detection. Actin was used as a loading control. C. Non-infected BL3 lymphocytes produce less H2O2 than infected TBL3 lymphocytes. D. Metabolomic analyses highlighting intracellular metabolites stemming from glycolytic activity associated with hexokinase, pyruvate kinase and lactate dehydrogenase in BL3 compared with TBL3 lymphocytes. TBL3 consume more glucose and produce more lactate than BL3, whereas pyruvate levels remain approximately equivalent. The intracellular lactate levels do not take into account lactate secreted into the culture medium. [file cmi0016-0269-sd1.tif]
